# Genome-wide association study for subclinical atherosclerosis in major arterial territories in the NHLBI's Framingham Heart Study

**DOI:** 10.1186/1471-2350-8-S1-S4

**Published:** 2007-09-19

**Authors:** Christopher J O'Donnell, L Adrienne Cupples, Ralph B D'Agostino, Caroline S Fox, Udo Hoffmann, Shih-Jen Hwang, Erik Ingellson, Chunyu Liu, Joanne M Murabito, Joseph F Polak, Philip A Wolf, Serkalem Demissie

**Affiliations:** 1 The National Heart, Lung, and Blood Institute's Framingham Heart Study, Framingham, MA, USA; 2 School of Medicine, Boston University, Boston, MA, USA; 3 School of Public Health, Boston University, Boston, MA, USA; 4 Department of Mathematics and Statistics, Boston University, Boston, MA, USA; 5 Cardiology Division, Department of Medicine, Massachusetts General Hospital, Boston, MA, USA; 6 Department of Radiology, Massachusetts General Hospital, Boston, MA, USA; 7 Endocrinology Division, Department of Medicine, Brigham and Women's Hospital, Harvard Medical School, Harvard University, Boston, MA, USA; 8 Department of Radiology, Tufts-New England Medical Center, Boston, MA, USA; 9 National Heart, Lung, and Blood Institute, National Institutes of Health, Bethesda, MD, USA

## Abstract

**Introduction:**

Subclinical atherosclerosis (SCA) measures in multiple arterial beds are heritable phenotypes that are associated with increased incidence of cardiovascular disease. We conducted a genome-wide association study (GWAS) for SCA measurements in the community-based Framingham Heart Study.

**Methods:**

Over 100,000 single nucleotide polymorphisms (SNPs) were genotyped (Human 100K GeneChip, Affymetrix) in 1345 subjects from 310 families. We calculated sex-specific age-adjusted and multivariable-adjusted residuals in subjects tested for quantitative SCA phenotypes, including ankle-brachial index, coronary artery calcification and abdominal aortic calcification using multi-detector computed tomography, and carotid intimal medial thickness (IMT) using carotid ultrasonography. We evaluated associations of these phenotypes with 70,987 autosomal SNPs with minor allele frequency ≥ 0.10, call rate ≥ 80%, and Hardy-Weinberg p-value ≥ 0.001 in samples ranging from 673 to 984 subjects, using linear regression with generalized estimating equations (GEE) methodology and family-based association testing (FBAT). Variance components LOD scores were also calculated.

**Results:**

There was no association result meeting criteria for genome-wide significance, but our methods identified 11 SNPs with p < 10^-5 ^by GEE and five SNPs with p < 10^-5 ^by FBAT for multivariable-adjusted phenotypes. Among the associated variants were SNPs in or near genes that may be considered candidates for further study, such as rs1376877 (GEE p < 0.000001, located in *ABI2*) for maximum internal carotid artery IMT and rs4814615 (FBAT p = 0.000003, located in *PCSK2*) for maximum common carotid artery IMT. Modest significant associations were noted with various SCA phenotypes for variants in previously reported atherosclerosis candidate genes, including *NOS3 *and *ESR1*. Associations were also noted of a region on chromosome 9p21 with CAC phenotypes that confirm associations with coronary heart disease and CAC in two recently reported genome-wide association studies. In linkage analyses, several regions of genome-wide linkage were noted, confirming previously reported linkage of internal carotid artery IMT on chromosome 12. All GEE, FBAT and linkage results are provided as an open-access results resource at http://www.ncbi.nlm.nih.gov/projects/gap/cgi-bin/study.cgi?id=phs000007.

**Conclusion:**

The results from this GWAS generate hypotheses regarding several SNPs that may be associated with SCA phenotypes in multiple arterial beds. Given the number of tests conducted, subsequent independent replication in a staged approach is essential to identify genetic variants that may be implicated in atherosclerosis.

## Background

Myocardial infarction, stroke and other atherosclerotic cardiovascular diseases comprise the leading cause of death for men and women in the U.S. [1], and will soon become the leading cause of death worldwide [2]. Atherosclerosis in the arterial wall precedes the onset of most cases of clinically apparent cardiovascular disease by decades, and subclinical atherosclerosis (SCA) is quite common in young and middle-aged persons [3-5]. SCA can be detected and quantified in major arteries, such as the carotid and coronary arteries, which provide essential blood flow to major organs, using noninvasive, high resolution imaging modalities. Such measures include peripheral arterial atherosclerosis detected by the ankle-brachial index (ABI); internal and common carotid intimal medial thickness (IMT) detected by B-mode ultrasound; and coronary artery calcium (CAC) and abdominal aortic calcium (AAC) deposits by multidetector computed tomography (MDCT). We have previously reported evidence for incomplete correlations between carotid IMT, CAC and aortic calcium [6]. Both genetic and environmental factors underlie interindividual variability in SCA, and significant heritability has been found for measures of SCA in the peripheral vasculature [7], carotid arteries [8], aorta [9], and coronary arteries [10]. Further, while there is evidence for partial correlation of major cardiovascular risk factors with atherosclerosis, multiple SCA measures, including ABI, carotid IMT, CAC, and AAC, have been shown to predict future risks for cardiovascular disease independent of risk factors [11-14].

While SCA measures are heritable, relatively little is known regarding the role of genetic variants in the interindividual variability in these quantitative measures of atherosclerosis. To date, results from candidate gene association studies for clinically apparent cardiovascular disease or subclinical disease have been inconsistent, though overviews of multiple studies seem to provide evidence for modest associations for variants in a number of candidate genes, such as *APOE *and *ACE *[8,15]. Genome-wide association studies (GWAS) using densely spaced single nucleotide polymorphisms (SNPs) provide a more comprehensive approach unconstrained by existing knowledge to test common genetic variation across the genome using high-throughput genotyping arrays. From GWAS, several previously unrecognized genes have been identified that may contribute to disease, including *CFH *and age-related macular degeneration [16], *INSIG2 *and obesity [17], *NOS1AP *with QT interval variation [18], and several genes including *TCF7L2 *[19] as well as *IGF2BP2* and *CDKA1* with diabetes mellitus [20-22]. Additionally, two other GWAS have identified an association with coronary heart disease of SNPs in a region of chromosome 9p21 [23,24] that was also associated with diabetes mellitus in three previous GWAS [20-22]. Of note, there was an association of this chromosome 9 region with the SCA measure of coronary artery calcium in one replication study [19,24].

With the availability of high resolution imaging of atherosclerosis phenotypes in specific arterial beds in community-based cohorts such as the Framingham Heart Study, GWAS analysis is now possible for quantitative SCA phenotypes in major arterial beds. Recently, over 100,000 single nucleotide polymorphisms (SNPs) were genotyped (Human 100K GeneChip, Affymetrix) in 1,345 subjects from 310 pedigrees in the Framingham Heart Study. The primary objective of this report is to present a brief summary of the results of this GWAS for SCA detected in the lower extremity arteries, carotid arteries, aorta and coronary arteries, by conduct of genetic association analyses and genetic linkage mapping. This report is one of a series of manuscripts from a collaborative project conducted by Framingham Heart Study investigators; the overall approach to this project's statistical genetic methods is summarized in an Overview manuscript that summarizes the 100K genome-wide association study [25].

## Materials and methods

### Study sample

Participants from the Offspring Cohort of the Framingham Heart Study who underwent one or more SCA measurements and genotyping with the Affymetrix 100K GeneChip are included in the study sample; genotyping was completed in a total of 1,345 subjects (1,084 Offspring cohort subjects). Original cohort subjects did not undergo the recently conducted MDCT or carotid IMT testing and as such were not included in this study. Of the 1,084 Offspring cohort subjects with genotyping, up to 984 participants with SCA phenotype information were analyzed. Details regarding selection of participants and genotyping are provided in the Overview [25].

### Phenotype definitions & methods

Determination of the clinical characteristics in Offspring participants reported here was obtained at study entry (baseline) and at each follow-up examination, including Offspring examinations 6 and 7. The methods of measurement of the covariates (blood pressure, body height and weight, lipids, diabetes mellitus, smoking and other clinical characteristics) used in these analyses have been previously described [26]. For imaging tests conducted between two examination cycles, covariates used in the analysis were obtained from the earlier examination cycle.

### Carotid ultrasonography for carotid IMT

Participants underwent carotid ultrasonography according to a previously reported standardized protocol at Offspring examination 6 (1995 to 1998) [27]. Imaging was conducted with a Toshiba SSH-140A imaging unit using a high resolution 7.5 MHz transducer for the common carotid artery, and a 5.0 MHz transducer for the internal carotid artery. Images were gated to an electrocardiogram; end-diastolic images were acquired. As previously described, correlation coefficients for the mean and maximum internal carotid artery were 0.83 and 0.84, respectively, based upon 25 readings by two separate readers [28].

All studies were recorded on optical disk and read according to a standardized protocol [29]. To quantify the degree of thickening of the carotid artery walls, the measures of IMT were summarized into two variables: one for common carotid artery and one for internal carotid artery. Mean and maximum wall thicknesses of the common and internal carotid artery were defined as the mean of the wall thickness or the mean of the maximum wall thickness for the near and far wall on the left and right sides. The number of available measurements for averaging ranged from 1–4 for the common carotid artery, and 1–8 for the internal carotid artery.

### MDCT for CAC and AAC

Measures of CAC and AAC were obtained between 2002 and 2005 in Offspring participants, between examinations 7 and 8. All eligible participants were imaged with an eight-slice multidetector computed tomography (Lightspeed Ultra, GE, Milwaukee, WI, USA) of the chest, as previously described [30], as well as the abdomen. Each subject underwent two chest CT scans and one abdominal scan that were performed using a sequential scan protocol with a slice collimation of 8 mm × 2.5 mm (120 KVp, 320/400 mA for .220 lbs body weight, respectively) during a single end-inspiratory breath hold (typical duration 18 s). Image acquisition (330 ms) was prospectively initiated at 50% of the cardiac cycle. For the abdominal scan, thirty contiguous 5 mm thick slices of the abdomen were acquired covering 150 mm above the level of S1. A calibration phantom (Image Analysis, Lexington, KY, USA) that contained rods of water and 75 and 150 mg/cm3 calcium hydroxyapatite, was placed underneath each subject.

Calcium measurements were performed on an offline workstation (Acquarius, Terarecon, San Matteo, CA, USA) by a trained technician. Scoring of coronary calcification has been previously described, and we reported excellent intra and inter reader reproducibility for the CAC measurements [30]. A calcified lesion in either the coronary arteries or in the aorta was defined as an area of at least three connected pixels with CT attenuation >130 Hounsfield Units using 3D connectivity criteria. A score for AAC (for the abdominal scan) and CAC (for each of the two chest scans) was calculated by multiplying the area of a calcified lesion with a weighted CT attenuation score dependent on the maximal CT attenuation (Hounsfield Units) within a lesion. In modification to the original Agatston Score that was originally developed for electron beam CT, we applied this algorithm to our MDCT scan protocol to score for CAC, as used in numerous previous studies [30], as well as for AAC.

### Ankle brachial index (ABI) for peripheral arterial disease

Ankle-brachial systolic blood pressure measurements were obtained at Offspring examinations 6 and 7 according to a standard protocol by trained technicians, as previously described [7]. Participants rested for a minimum of five minutes in the supine position on the examining table prior to blood pressure measurement. Blood pressure cuffs were applied to bare ankles with the midpoint of the bladder over the posterior tibial artery approximately three centimeters above the medial malleolus. Systolic blood pressure was measured using an 8 megahertz Doppler pen probe and an ultrasonic Doppler flow detector (Parks Medical Electronics, Inc.). For each limb, (right and left arms, right and left ankles) the cuff was inflated quickly to the maximal inflation level and deflated at a rate of 2 mmHg per second until the systolic blood pressure became audible. All limb blood pressures were repeated in reverse order. If the initial and repeat blood pressures differed by more than 10 mmHg at any one site, a third measurement was taken. Measurement was taken from the dorsalis pedis artery only if the posterior tibial pulse could not be located by palpation or with the Doppler probe [7].

The ABI is defined as the ratio of the average systolic blood pressure in the ankle divided by the average systolic blood pressure in the arm. The higher arm mean was used to calculate the ankle-brachial index for each leg. The lower of the two ankle-brachial index measurements was used for analysis.

### Genotyping methods

Details of the genotyping methods are available in the Overview [25]. Briefly, 112,990 SNPs on the Affymetrix 100K chip were genotyped using DNA from family members of the Framingham Heart Study. For this report, SNPs were excluded for the following reasons: minor allele frequency ≤10% (n = 38,062); genotypic call rate ≤80% (n = 2346); Hardy Weinberg Equilibrium p-value ≤ 0.001(n = 1,595), leaving 70,987 SNPs available for analysis [25]. Results for all SNPs are reported on the open-access results website.

### Statistical analysis methods

In total, of the 1,084 Offspring cohort subjects with genotyping, 673 to 984 participants with analyzable phenotype information were available for analysis. Residuals were created from multiple linear regression models in all subjects with the SCA measures, regardless of whether they were genotyped or not, to adjust phenotypes for covariates; these residuals were created in women and men separately. Covariate adjustment for blood pressure and/or hypertension was performed is several different ways. For ABI, a dichotomous measure for hypertension (systolic blood pressure > 140 or diastolic blood pressure > 90 or on treatment) was used. For CAC and AAC, systolic blood pressure and use of anti-hypertensive treatment were used as covariates. For carotid IMT, treatment-adjusted systolic blood pressure was used. As previously described, systolic blood pressures for those on treatment are imputed to estimate what these values would be if the subject were not on treatment [31]. Ranked normalized deviates, created from the standardized residuals from the regression models, were used in the genetic analyses. For each sex, SCA phenotypes were age-adjusted and multivariable-adjusted; details of the covariates included in the multivariable adjustment for each phenotype are presented in Table 1.

**Table 1:** Phenotype distribution, examination cycle, and numbers of participants with subclinical atherosclerosis phenotypes

Subclinical atherosclerosis measure	Phenotype Variable name	N	Offspring, exam	Covariate adjustment
Ankle-brachial index	RANKLEBI6	984	Offspring, 6	Age, sex-specific
	RANKLEBI6MV	984	Offspring, 6	Age, smoking, diabetes, hypertension, total cholesterol/HDL ratio, log triglyceride, sex-specific
	RANKLEBI7	982	Offspring, 7	Age, sex-specific
	RANKLEBI7MV	982	Offspring, 7	Age, smoking, diabetes, hypertension, total cholesterol/HDL ratio, log triglyceride, sex-specific
Maximum carotid artery bulb IMT	RNKCAROTBULBAS6	959	Offspring, 6	Age, sex-specific
	RNKCAROTBULBMV6	951	Offspring, 6	Age, treatment-adjusted systolic blood pressure, BMI, cigarettes per day, HDL cholesterol, total cholesterol, diabetes, log triglyceride, and in women, menopausal status, hormone therapy, sex-specific
Maximum common carotid artery IMT	RNKCAROTCCAMAXAS6	978	Offspring, 6	Age, sex-specific
	RNKCAROTCCAMAXMV6	969	Offspring, 6	Age, treatment-adjusted systolic blood pressure, BMI, cigarettes per day, HDL cholesterol, total cholesterol, diabetes, log triglyceride, and in women, menopausal status, hormone therapy, sex-specific
Mean common carotid artery IMT	RNKCAROTCCAMEANAS6	978	Offspring, 6	Age, sex-specific
	RNKCAROTCCAMEANMV6	969	Offspring, 6	Age, treatment-adjusted systolic blood pressure, BMI, cigarettes per day, HDL cholesterol, total cholesterol, diabetes, log triglyceride, and in women, menopausal status, hormone therapy, sex-specific
Maximum internal carotid artery IMT	RNKCAROTICAMAXAS6	888	Offspring, 6	Age, sex-specific
	RNKCAROTICAMAXMV6	880	Offspring, 6	Age, treatment-adjusted systolic blood pressure, BMI, cigarettes per day, HDL cholesterol, total cholesterol, diabetes, log triglyceride, and in women, menopausal status, hormone therapy, sex-specific
Mean internal carotid artery IMT	RNKCAROTICAMEANAS6	888	Offspring, 6	Age, sex-specific
	RNKCAROTICAMEANMV6	880	Offspring, 6	Age, treatment-adjusted systolic blood pressure, BMI, cigarettes per day, HDL cholesterol, total cholesterol, diabetes, log triglyceride, and in women, menopausal status, hormone therapy, sex-specific
Maximum carotid artery stenosis	RNKCAROTSTENAS6	977	Offspring, 6	Age, sex-specific
	RNKCAROTSTENMV6	968	Offspring, 6	Age, treatment-adjusted systolic blood pressure, BMI, cigarettes per day, HDL cholesterol, total cholesterol, diabetes, log triglycerides, menopausal status, hormone therapy, sex-specific
Mean abdominal aortic calcification	RESMDCTAACAS7	675	Offspring, 7	Age, sex-specific
	RESMDCTAACMV7	673	Offspring, 7	Age, BMI, current cigarette smoking status, diabetes, systolic blood pressure, anti-HTN therapy, total-to-HDL-C ratio, lipid therapy, sex-specific
Mean coronary artery calcification	RESMDCTCACAS7	680	Offspring, 7	Age, sex-specific
	RESMDCTCACMV7	678	Offspring, 7	Age, BMI, current cigarette smoking status, Diabetes, systolic blood pressure, anti-HTN therapy, total-to-HDL cholesterol ratio, lipid therapy, sex-specific
Maximum coronary artery calcification	RESMDCTCACMAXAS7	680	Offspring, 7	Age, sex-specific
	RESMDCTCACMAXMV7	678	Offspring, 7	Age, BMI, current cigarette smoking status, diabetes, systolic blood pressure, anti-HTN therapy, total-to-HDL cholesterol ratio, lipid therapy, sex-specific

Abbreviations: IMT = intimal medial thickness; MDCT = multidetector Computed Tomography; AAC = abdominal aortic calcification; CAC = coronary artery calcification; HDL = high density lipoprotein; BMI = body mass index; anti-HTN therapy = drug treatment for hypertension; lipid therapy = drug treatment for hyperlipidemia.

All association analyses were performed using either generalized estimating equations (GEE) or family-based association testing (FBAT). We evaluated associations of the SCA phenotypes using an additive genetic model with 70,987 SNPs meeting criteria above. Details regarding these analytic methods are provided in the Overview [25]. Only results of multivariable-adjusted SCA phenotypes are displayed in Table 2. Linkage analysis was performed using variance components methods on a subset of the 100K chip SNPs in linkage equilibrium and Marshfield STR markers that were previously obtained [25].

**Table 2:** 25 Most significant results for multivariable-adjusted subclinical atherosclerosis measures in multiple arterial territories by GEE (2a), FBAT (2b) and linkage (2c) analyses

2a. Most significant results for GEE analyses for multivariable-adjusted subclinical atherosclerosis measures								
**GEE rank**	**Phenotype**	**SNP**	**Chr**	**Physical location**	**GEE P-value**	**FBAT P-value**	**Gene position**	**Gene symbol**

1	Internal carotid artery IMT	rs1376877	2	204,097,596	3.8 × 10^-7^	0.15	IN	*ABI2*
3	Coronary artery calcification	rs2390582	1	90,655,928	1.4 × 10^-6^	0.34	OUT	
4	Abdominal aortic calcification	rs3849150	10	49,779,229	1.6 × 10^-6^	4.0 × 10^-3^	NEAR	*LRRC18*
6	Ankle brachial index	rs2896103	5	13,817,419	4.5 × 10^-6^	0.01	IN	*DNAH5*
7	Coronary artery calcification	rs10483853	14	72,826,052	6.1 × 10^-6^	1.7 × 10^-3^	IN	*NUMB*
8	Common carotid artery IMT	rs1400544	1	186,593,061	6.2 × 10^-6^	4.7 × 10^-4^	OUT	
9	Ankle brachial index	rs7715811	5	13,822,974	6.4 × 10^-6^	0.02	IN	*DNAH5*
10	Coronary artery calcification	rs10507130	12	100,256,422	6.7 × 10^-6^	1.6 × 10^-3^	IN	*DRIM*
11	Ankle brachial index	rs1320267	4	127,290,897	6.9 × 10^-6^	0.03	OUT	
13	Ankle brachial index	rs1350445	11	91,591,802	8.5 × 10^-6^	0.07	OUT	
14	Ankle brachial index	rs1502050	5	13,832,743	8.7 × 10^-6^	0.02	IN	*DNAH5*
15	Coronary artery calcification	rs10519394	4	137,962,214	1.1 × 10^-5^	0.03	OUT	
16	Internal carotid artery IMT	rs683366	18	52,370,975	1.2 × 10^-5^	0.01	NEAR	*TXNL1*
19	Abdominal aortic calcification	rs2850711	18	59,938,018	1.5 × 10^-5^	0.05	IN	*C18orf20*
20	Internal carotid artery IMT	rs601746	10	96,932,273	1.6 × 10^-5^	0.22	NEAR	*C10orf129*
21	Ankle brachial index	rs1905155	5	113,301,097	1.6 × 10^-5^	5.1 × 10^-3^	OUT	
22	Ankle brachial index	rs10501784	11	91,592,192	1.6 × 10^-5^	0.30	OUT	
23	Ankle brachial index	rs10493529	1	74,335,469	1.9 × 10^-5^	0.098	NEAR	*LRRC44*, *FPGT*
24	Common carotid artery IMT	rs28207	5	13,267,852	1.9 × 10^-5^	2.1 × 10^-4^	OUT	
25	Abdominal aortic calcification	rs8094641	18	59,873,880	2.0 × 10^-5^	0.04	NEAR	*C18orf20*
26	Ankle brachial index	rs2949535	18	26,111,783	2.0 × 10^-5^	7.5 × 10^-4^	OUT	
27	Coronary artery calcification	rs2270861	12	100,238,849	2.0 × 10^-5^	6.8 × 10^-3^	IN	*DRIM*
28	Common carotid artery IMT	rs853406	6	4,002,159	2.2 × 10^-5^	1.9 × 10^-3^	IN	*PRPF4B*
29	Common carotid artery IMT	rs853407	6	4,002,232	2.2 × 10^-5^	2.5 × 10^-3^	IN	*PRPF4B*
30	Common carotid artery IMT	rs1581413	3	158,532,867	2.2 × 10^-5^	1.8 × 10^-3^	IN	*VEPH1*

**2b. Most significant results for FBAT analyses for multivariable-adjusted subclinical atherosclerosis measures**								

**FBAT rank**	**Phenotype**	**SNP**	**Chr**	**Physical location**	**GEE P-value**	**FBAT P-value**	**Gene position**	**Gene symbol**

1	Common carotid artery IMT	rs4814615	20	17,305,573	4.9 × 10^-5^	3.4 × 10^-6^	IN	*PCSK2*
2	Common carotid artery IMT	rs6053733	20	5,763,074	7.2 × 10^-3^	3.7 × 10^-6^	IN	*C20orf196*
3	Ankle brachial index	rs10499903	7	90,504,268	1.9 × 10^-3^	4.1 × 10^-6^	NEAR	*PFTK1*, *FZD1*
4	Ankle brachial index	rs9302997	17	71,723,439	4.8 × 10^-3^	5.1 × 10^-6^	IN	*RNF157*
5	Ankle brachial index	rs590183	4	23,733,535	0.01	8.6 × 10^-6^	OUT	
7	Ankle brachial index	rs6832344	4	23,733,805	9.9 × 10^-3^	1.0 × 10^-5^	OUT	
8	Abdominal aortic calcification	rs1023568	2	191,340,948	0.09	1.2 × 10^-5^	IN	*NAB1*
9	Common carotid artery IMT	rs2214959	12	124,674,521	0.01	1.5 × 10^-5^	NEAR	*TMEM132B*
10	Ankle brachial index	rs9285151	13	42,712,236	0.06	1.7 × 10^-5^	IN	*ENOX1*
11	Ankle brachial index	rs1381632	4	88,926,163	0.36	1.8 × 10^-5^	NEAR	*DMP1*
12	Coronary artery calcification	rs4461066	16	51,469,110	8.7 × 10^-3^	3.1 × 10^-5^	OUT	
15	Coronary artery calcification	rs367421	20	15,202,689	2.5 × 10^-3^	3.6 × 10^-5^	IN	*C20orf133*
16	Internal carotid artery IMT	rs9323431	14	62,638,241	0.24	3.6 × 10^-5^	IN	*KCNH5*
17	Common carotid artery IMT	rs1997463	3	54,782,280	0.01	3.8 × 10^-5^	IN	*CACNA2D3*
19	Common carotid artery IMT	rs304409	18	1,096,342	1.8 × 10^-4^	4.6 × 10^-5^	OUT	
21	Internal carotid artery IMT	rs1349008	3	76,804,103	0.23	4.9 × 10^-5^	OUT	
22	Ankle brachial index	rs2251671	8	40,501,973	1.3 × 10^-4^	4.9 × 10^-5^	NEAR	*ZMAT4*
23	Abdominal aortic calcification	rs243030	2	60,518,222	3.1 × 10^-5^	5.6 × 10^-5^	OUT	
24	Abdominal aortic calcification	rs321967	7	77,959,200	4.7 × 10^-4^	6.0 × 10^-5^	IN	*MAGI2*
25	Ankle brachial index	rs6569792	6	132,736,444	0.56	6.4 × 10^-5^	IN	*MOXD1*
26	Abdominal aortic calcification	rs4985741	17	17,028,605	7.2 × 10^-3^	6.6 × 10^-5^	IN	*MRIP*
27	Ankle brachial index	rs7995026	13	42,751,550	6.9 × 10^-4^	6.9 × 10^-5^	IN	*ENOX1*
28	Coronary artery calcification	rs10520541	4	184,055,754	0.08	7.0 × 10^-5^	IN	*AK001336*
29	Internal carotid artery IMT	rs2113945	15	29,111,823	0.09	7.2 × 10^-5^	IN	*TRPM1*
30	Common carotid artery IMT	rs6944400	7	131,389,740	0.12	8.2 × 10^-5^	OUT	

**2c. Genomic regions with LOD > 2.0 for linkage analyses for multivariable-adjusted subclinical atherosclerosis measures**								

**Rank**	**Phenotype**	**Marker**	**Chr**	**Physical location**	**LOD score 1.5 interval bounds**		**Maximum LOD**	

1	Internal carotid artery IMT	ATA29A06	12	128,922,618	127,583,749	129,277,512	5.05	
2	Internal carotid artery IMT	rs959987	12	127,817,680	126,600,362	129,277,512	4.78	
3	Internal carotid artery IMT	rs1180937	1	54,903,665	45,417,474	56,950,040	4.23	
8	Abdominal aortic calcification	rs17030524	12	99,640,364	97,320,770	102,339,523	3.51	
6	Coronary artery calcification	SNP_A-1679277	6	149,300,408	136,476,887	154,228,170	2.81	
7	Ankle brachial index	rs394884	2	151,954,268	142,801,622	168,477,710	2.73	
9	Internal carotid artery IMT	rs300574	4	124,681,343	118,755,997	131,657,093	2.49	
10	Internal carotid artery IMT	rs714647	15	99,870,157	96,719,695	100,152,332	2.49	
12	Coronary artery calcification	rs1106679	14	102,555,405	91,636,556	106,312,036	2.33	
13	Abdominal aortic calcification	rs1454183	2	13,935,308	3,238,478	20,119,479	2.31	
15	Coronary artery calcification	rs7000744	8	140,003,669	136,360,837	146,039,126	2.23	
16	Ankle brachial index	rs10483084	21	42,268,393	40,113,290	45,025,009	2.12	
17	Internal carotid artery IMT	rs1948685	5	21,064,843	10,288,046	31,560,322	2.11	
19	Internal carotid artery IMT	rs10832008	11	13,232,905	6,384,682	19,637,706	2.09	

Abbreviations: SNP = single nucleotide polymorphism; Chr = chromosome; GEE = generalized estimating equations; FBAT = family based association testing; LOD = logarithm of the odds; IMT = intimal medial thickness;. Gaps appear in the rank order for markers that appear more than once. In the column entitled “Phenotype”, 'internal carotid artery IMT' denotes either mean or maximum internal carotid IMT, 'common carotid artery IMT' denotes either mean or maximum common carotid IMT, 'coronary artery calcification' denotes either mean CAC or max CAC, and 'ankle brachial index' denotes and ankle brachial index from either examination cycle 6 or cycle 7. For proximity to known genes (Table columns nine and ten), “IN” refers to a SNP within a protein-coding gene intron or exon, “OUT” refers to a SNP greater than 60 kb away from a protein-coding gene gene, and “NEAR” refer to a SNP within 60 kb of a protein-coding gene.

We further sought to identify SNPs with consistent associations with phenotypes within measurement groups using GEE and FBAT analyses. SNPs were selected based on associations with multiple correlated traits in both population- and family-based tests. We evaluated the following five phenotypic subgroups using sex-specific age-adjusted and multivariable-adjusted residuals (Table 1): ABI, common carotid artery IMT, internal carotid artery IMT, AAC, and CAC. For each SNP we calculated the proportion of phenotypes significantly associated with the SNP with *P *< 0.01 in both GEE and FBAT. For instance, for ABI, we evaluated GEE and FBAT results for both age-sex-adjusted and multivariable-adjusted measures of ankle-brachial index from both examination cycle 6 and 7 (in Table 1, these variables are named RANKLEBI6, RANKLEBI6MV, RANKLEBI7, and RANKLEBI7MV). Results are displayed in Table 3 of the top 5 SNPs with the highest proportions of significantly (*P *< 0.01) associated phenotypes. For identical proportions of significant phenotypes, SNPs were additionally ranked by the logarithm of the mean of GEE p-values.

**Table 3:** Five most significant association results using FBAT and GEE within phenotype clusters for each of five multivariable-adjusted subclinical atherosclerosis measures

Phenotype group	Rank GEE/FBAT	SNP	Chr	Physical location	%Phenotypes P < 0.01 for GEE & FBAT	GEE P-value geometric mean	MAF	Gene position	Gene symbol
**Ankle brachial index**	1	rs7989017	13	25,164,878	100	4.7 × 10^-3^	0.22	IN	*ATP8A2*
	2	rs7546903	1	6,870,538	75	1.7 × 10^-3^	0.27	IN	*CAMTA1*
	3	rs6135095	20	1,422,405	75	3.0 × 10^-3^	0.14	NEAR	*SIRPB2*
	4	rs1875517	3	118,790,257	75	3.4 × 10^-3^	0.44	OUT	
	5	rs6507763	18	43,310,669	75	3.5 × 10^-3^	0.14	OUT	

**Common carotid artery IMT**	1	rs1039610	18	74,049,795	100	4.9 × 10^-5^	0.32	OUT	
	2	rs1587893	18	74,039,913	100	5.8 × 10^-5^	0.31	OUT	
	3	rs28207	5	13,267,852	100	8.1 × 10^-5^	0.37	OUT	
	4	rs4814615	20	17,305,573	100	1.3 × 10^-4^	0.16	IN	*PCSK2*
	5	rs2470209	17	28,263,442	100	1.9 × 10^-4^	0.39	NEAR	*MYO1D*

**Internal carotid artery IMT**	1	rs933890	12	106,941,389	100	6.6 × 10^-4^	0.22	OUT	
	2	rs8075776	17	36,408,189	100	9.8 × 10^-4^	0.16	NEAR	*KRTAP1-1*, *KRTAP1-3*
	3	rs252984	5	106,780,236	100	1.0 × 10^-3^	0.16	IN	*EFNA5*
	4	rs10490889	3	6,257,028	100	1.6 × 10^-3^	0.36	OUT	
	5	rs10516308	4	16,780,451	100	3.3 × 10^-3^	0.41	OUT	

**Coronary artery calcification**	1	rs10483853	14	72,826,052	100	9.8 × 10^-6^	0.19	IN	*NUMB*
	2	rs10507130	12	100,256,422	100	1.3 × 10^-5^	0.22	IN	*DRIM*
	3	rs9321354	6	132,946,880	100	3.4 × 10^-5^	0.12	NEAR	*TAAR8*, *TAAR6*, *TAAR5*
	4	rs220457	17	27,126,748	100	3.8 × 10^-4^	0.25	OUT	
	5	rs10505182	8	113,337,274	100	5.2 × 10^-4^	0.23	IN	*CSMD3*

Abbreviations: SNP = single nucleotide polymorphism; Chr = chromosome; GEE = generalized estimating equations; FBAT = family based association testing; IMT = intimal medial thickness; MAF = minor allele frequency.

Column Definitions: For each Phenotype Group (Table column one), a cluster analysis is conducted for several phenotype variables defined in **Table 1**. For “Ankle brachial index,” GEE and FBAT results were evaluated for RANKLEBI6, RANKLEBI6MV, RANKLEBI7, and RANKLEBI7MV; for “Common carotid artery IMT,” RNKCAROTCCAMAXAS6, RNKCAROTCCAMAXMV6, RNKCAROTCCAMEANAS6, and RNKCAROTCCAMEANMV6; for “Internal carotid artery IMT,” RNKCAROTICAMAXAS6, RNKCAROTICAMAXMV6, RNKCAROTICAMEANAS6, and RNKCAROTICAMEANMV6; and for “Coronary artery calcification,” RESMDCTCACMAXMV7 and RESMDCTCACMV7. The percent of phenotypes in the cluster analysis for which there was a p < 0.01 by both GEE and FBAT analyses is shown in Table column five. The geometric mean p-value is shown in Table column six for GEE associations for all phenotypes in the cluster analyses. For proximity to known genes (Gene position, Table column eight), “IN” refers to a SNP within a protein-coding gene intron or exon, “OUT” refers to a SNP greater than 60 kb away from a protein-coding gene gene, and “NEAR” refer to a SNP within 60 kb of a protein-coding gene.

## Results

Ten SCA atherosclerosis measures were studied (Table 1). For each SCA measurement, sex-specific, age-adjusted phenotypes as well as age- and multivariable-adjusted phenotypes were evaluated. Measures for ABI and carotid IMT phenotypes were available in 880–984 participants, whereas measures for MDCT phenotypes were available in 673–680 participants.

Tables 2a, 2b and 2c provides a summary of the most significant findings in GEE, FBAT and linkage analyses, respectively, across a number of selected multivariable-adjusted phenotypes described in Table 1. Full-disclosure of all results for all associations can be found at http://www.ncbi.nlm.nih.gov/projects/gap/cgi-bin/study.cgi?id=phs000007. The top 25 most statistically significant associated SNPs in GEE analyses are presented in Table 2a. There were 11 SNPs with p < 10^-5 ^in GEE analyses. The top three associated SNPs are rs1376877 (p < 1*10^-6^, located in *ABI2*) associated with maximum internal carotid IMT, rs2390582 (p = 1*10^-6^, not located near a known gene) associated with maximum CAC score, and rs3849150 (p = 2*10^-6^, located near LRRC18) associated with AAC score. The top 25 most significantly associated SNPs in FBAT analyses are shown in Table 2b. There were 5 SNPs with p < 10^-5 ^by FBAT. The top three associated SNPs by FBAT are rs4814615 (p = 3*10^-6^, located in *PCSK2*) associated with maximum common carotid IMT, rs6053733 (p = 4*10^-6^, located near *FLJ25067*) associated with mean common carotid IMT, and rs10499903 (p = 4*10^-6^, located near *PFTK1*) associated with ABI.

When we examined below the top 25 associations on our list of nominally significant GEE results for various SCA phenotypes, there were significant associations with multivariable-adjusted CAC for several SNPs on chromosome 9, including three SNPs (rs10511701, rs1556516 and rs1537371; p-values for association 1.1 × 10^-4^, 8.8 × 10^-5^, and 1.7 × 10^-4^, respectively) lying within a 15 kB region implicated in recent GWAS's for coronary heart disease. Similar associations were noted for age-and sex-adjusted residuals for CAC.

Results for all multipoint LOD scores > 2.0 are displayed in Table 2c. There were four LOD score results exceeding 3.0 and ten additional LOD score results exceeding 2.0. Results for linkage of internal carotid artery IMT phenotypes to chromosome 12 and 1 (the top three LOD score results) and for chromosome 11 are consistent with findings from our previous report [32]. Upon further inspection of the list of associations with p < 0.01 for each of the phenotypes, SNPs in or near biologically plausible genes were noted, including: fibroblast growth factor (*FGF1*) for AAC, adrenergic, beta-2-, receptor (*ADRB2*) for CAC, myocyte enhancer factor 2C (*MEF2C*) and thrombospondin 2 (*THBS2*) for common carotid artery IMT, and cAMP-specific phosphodiesterase 4D (*PDE4D*) for ankle brachial index.

In Table 3, we provide a summary of the top five associated SNPs for each of four phenotypic categories – ABI, common carotid artery IMT, internal carotid artery IMT, and CAC. SNPs were rank ordered first according to percent of phenotypes with p < 0.01 for GEE and FBAT associations and second by geometric mean of GEE p-values. A number of SNPs were common to the top 25 associated SNPs by GEE or FBAT reported in Tables 2a and 2b, respectively, including rs28207 and rs4814615 for common carotid IMT and rs10483853 and rs10507130 for CAC.

We further examined associations with SNPs in or near regions of 37 candidate genes previously reported to have been associated with SCA or overt coronary heart disease. Of these genes, there were 28 genes with SNPs on the 100K array that were in or nearby (within 60 kb of) the gene. In Table 4, we report significant associations for SNPs in or near nine of these 28 candidate genes with p < 0.01 in GEE or FBAT analyses or p < 0.05 in both GEE and FBAT analyses. Associations were noted with one or more SCA measure for: *CCR5*, *FGB*, *ESR1*, *IL6*, *NOS3*, *TNFRSF11*, *ADM*, *CD44*, *and CCL2 *(Table 4).

**Table 4:** SNPs in or near previously reported candidate genes for coronary heart disease or subclinical atherosclerosis for multivariable-adjusted SCA phenotypes: SNPs with p < 0.01 in GEE or FBAT analyses, or p < 0.05 in both GEE and FBAT analyses.

Chr	Gene	N SNPs within 60 kb	SNP from Affy 100K Array	MAF	Physical Location	Phenotype	Lowest P-value	
							**GEE**	**FBAT**

3	*CCR5*	1/6	rs3762823	0.25	46,371,620	Common carotid artery IMT	0.02	0.04
						Common carotid artery IMT	0.05	0.04
4	*FGB*	1/7	rs871540	0.22	155,766,635	Abdominal aortic calcification	0.11	3.0 × 10^-3^
6	*ESR1*	8/18	rs3866461	0.12	152,238,835	Coronary artery calcification	5.4 × 10^-3^	0.11
						Coronary artery calcification	3.4 × 10^-3^	0.12
			rs3853250	0.45	152,252,014	Ankle brachial index	0.05	0.04
			rs3853251	0.34	152,252,870	Ankle brachial index	0.03	0.02
			rs722208	0.27	152,414,999	Coronary artery calcification	0.54	5.7 × 10^-3^
						Coronary artery calcification	0.57	5.0 × 10^-3^
			rs3798573	0.11	152,481,476	Internal carotid artery IMT	6.4 × 10^-3^	0.78
			rs3778099	0.10	152,510,689	Common carotid artery IMT	0.82	6.9 × 10^-3^
			rs2813563	0.19	152,538,601	Abdominal aortic calcification	0.02	0.03
			rs725467	0.19	152,539,833	Abdominal aortic calcification	0.02	0.03
7	*IL6*	2/9	rs1581498	0.45	22,681,483	Coronary artery calcification	0.16	7.7 × 10^-3^
						Coronary artery calcification	0.04	3.3 × 10^-3^
			rs10240716	0.27	22,765,225	Ankle brachial index	0.01	0.04
7	*NOS3*	2/3	rs768403	0.38	150,297,894	Abdominal aortic calcification	2.0 × 10^-3^	0.02
			rs7812088	0.12	150,357,477	Internal carotid artery IMT	0.05	5.5 × 10^-3^
						Internal carotid artery IMT	0.05	0.01
8	*TNFRSF11*	1/9	rs10505346	0.21	120,033,024	Ankle brachial index	0.04	0.05
11	*ADM*	3/5	rs10500724	0.44	10,258,592	Ankle brachial index	2.0 × 10^-4^	5.0 × 10^-4^
			rs4444073	0.47	10,288,240	Ankle brachial index	3.3 × 10^-3^	5.0 × 10^-4^
			rs4444073			Ankle brachial index	0.01	0.04
11	*CD44*	10/24	rs1365057	0.22	35,066,251	Coronary artery calcification	0.05	8.9 × 10^-3^
						Coronary artery calcification	0.05	7.7 × 10^-3^
			rs353589	0.21	35,077,802	Ankle brachial index	9.0 × 10^-3^	0.08
						Abdominal aortic calcification	7.4 × 10^-3^	0.10
			rs353639	0.21	35,140,940	Ankle brachial index	8.3 × 10^-3^	0.03
						Ankle brachial index	3.1 × 10^-3^	0.04
			rs353638	0.21	35,141,103	Ankle brachial index	5.6 × 10^-3^	0.05
						Ankle brachial index	4.2 × 10^-3^	0.06
			rs353636	0.21	35,141,306	Ankle brachial index	7.6 × 10^-3^	0.05
						Ankle brachial index	4.2 × 10^-3^	0.06
			rs353635	0.46	35,141,399	Ankle brachial index	0.09	4.6 × 10^-3^
			rs353631	0.20	35,144,144	Ankle brachial index	4.8 × 10^-3^	0.06
						Ankle brachial index	4.5 × 10^-3^	0.15
			rs1467558	0.21	35,186,249	Coronary artery calcification	0.03	0.02
						Coronary artery calcification	0.03	0.02
			rs10488813	0.13	35,225,530	Abdominal aortic calcification	8.8 × 10^-3^	0.48
			rs7115246	0.46	35,243,054	Ankle brachial index	7.1 × 10^-3^	9.4 × 10^-3^
17	*CCL2*	1/7	rs1024612	0.41	29,573,469	Abdominal aortic calcification	3.6 × 10^-3^	0.22

Abbreviations: SNP = single nucleotide polymorphism; Chr = chromosome; GEE = generalized estimating equations; FBAT = family based association testing; IMT = intimal medial thickness; MAF = minor allele frequency.

Of 37 candidate genes considered, there were 28 genes covered by the 100K array and meeting exclusion criteria, of which nine genes had a significantly associated SNP(s) with either p < 0.01 for GEE, p < 0.01 for FBAT, or p < 0.05 for both GEE and FBAT. Genes without significant associations meeting these criteria included: *F5*, *MTHFR*, *REN*, *APOB*, *CX3CR1*, *GATA2*, *EDN1*, *CTGF*, *VEGF*, *PON1*, *MMP3*, *SCARB1*, *ALOX5AP*, *CETP*, *ITGB3*, *NOS2A*, *APO3*, *and MMP9*. However, it should be noted that 100K SNP coverage of any given gene region may be insufficient to exclude real associations. Better coverage may be afforded by newer, more dense SNP arrays. Only SNPs within the introns or exons of the candidate gene or no greater than 60 kb away from the candidate gene were considered for Table 4 (denominator of Column 3).

## Discussion

In this manuscript, we report the principle findings for a GWAS of SNPs from a 100K Affymetrix scan with SCA phenotypes in multiple major arterial beds. A conservative Bonferroni correction using this number of tests (0.05/1,000,000) would yield an approximate threshold of genome-wide significance to be 5*10^-8^, and none of our association findings meet these criteria for genome-wide association., However, we did note a number of associations with p < 10^-5 ^after rank-ordering SNP associations using GEE and/or FBAT analysis. While we expect that many of these genotype-phenotype associations may be false positives, the SNPs identified in this manner are nonetheless hypothesized to include SNPs that merit further follow-up. To our knowledge, this is the first report of a GWAS using SCA phenotypes.

The first replicated GWAS findings for clinically apparent coronary heart disease were recently reported by two independent groups, identifying an association with SNPs in a region of chromosome 9p21 [23,24]. Interestingly, there was an association of this chromosome 9 region with CAC in one replication study [24], and this same chromosome 9 region was associated with diabetes mellitus in three other recent GWAS [20-22]. When we examined our GEE results for various SCA phenotypes, there were nominally significant associations with multivariable-adjusted CAC for several SNPs on chromosome 9 lying within a 15 kB region implicated in the recent GWAS's for coronary heart disease. While these SNPs were not our top-ranked SNPs, their specific association with coronary artery atherosclerosis (CAC) phenotypes likely represent strong evidence of replication in light of the recent GWAS.

Because there may be distinct genetic determination for atherosclerosis occurring in individual vascular beds (e.g., carotid arteries versus coronary arteries versus aorta versus peripheral arteries), we further examined for consistency of association of specific SNPs within five trait groups – ABI, common carotid artery IMT, internal carotid artery IMT, AAC and CAC. By this method of ranking associations, we identified distinct sets of SNPs, for which there was consistency of overlap with top SNP results by both GEE and FBAT (Table 3). There is evidence for partial correlation between SCA measures [6], and it is interesting to note that there were some SNPs for which there were nominally significant associations with more than one SCA measure. For example, rs10263213 is associated with both multivariable adjusted CAC (p = 0.001) and multivariable adjusted wall thickness of the carotid bulb (p = 0.013). Further research is warranted to determine whether SNPs showing association with two or more SCA measures are more likely to replicate in follow-up studies. Again, because no results from these approaches met criteria for genome-wide association, further validation studies will be required to confirm such associations.

An extensive literature exists for prior studies of candidate gene variation associated with clinical atherosclerotic cardiovascular disease, in particular coronary heart disease [15], as well as SCA, particularly carotid IMT [8]. While many associations are not consistently noted from study to study, a review of the available studies reveals a modest overall association of clinical cardiovascular disease or SCA with variants in candidate genes, such as *APOE*, *ACE*, and *NOS3 *[15]. More recently, a number of screens of larger numbers of variants have revealed new candidate gene hypotheses, such as *ALOX5AP *[33,34] and *LGALS2 *[35]. When we examined for associations between SCA and SNPs on the 100K chip that reside in or near some previously studied candidate genes, we confirmed a number of modestly significant SNP associations with SCA phenotypes (Table 4). These and other associations between SNPs and various SCA measures may be viewed as supportive though not strongly confirmatory of prior hypotheses. However, it should be noted that 100K SNP coverage of any given gene region may be insufficient to exclude real associations. Better coverage may be afforded by newer, more dense SNP arrays. Nevertheless, these data suggest that GWAS may be used to contribute substantial information to the literature on the presence and strength of previously reported "candidate gene" associations, including those identified from other GWAS, as well as for unbiased searches for novel variants.

Our study was conducted in a moderate-sized, well-characterized community-based sample, and the conduct of SCA imaging was conducted without ascertainment for prior cardiovascular disease. Strengths and limitations of the GWAS in this sample using a 100K screen are discussed in the Overview [25]. A strength of this particular study is the substantial heritability of SCA phenotypes determined by high resolution imaging conducted in a reproducible manner. While these SCA phenotypes represent state of the art, non-invasive imaging in populations, it must be acknowledged that the available modalities allow a focus on only fixed anatomic components (e.g., calcific plaque or IMT) rather than dynamic or metabolically active components. Moreover, the occurrence and distribution of these modalities may differ by race or environmental background. Thus, our results may not extrapolate to non-white populations.

In conclusion, using a GWAS unconstrained by existing knowledge, we have identified new candidate SNP association hypotheses and further confirm some existing candidate gene and candidate SNP hypotheses. In particular, the evidence for association of several SNPs with CAC in the region of chromosome 9 that was recently reported to be associated with CHD or CAC in tens of thousands of subjects [23,24] provides evidence that we were able to identify true associations. These findings provide evidence for the exciting promise of GWAS of SCA to identify novel genetic variants underlying atherosclerosis within specific arterial territories or across multiple arterial territories. In this manuscript and in the accompanying web-posted results http://www.ncbi.nlm.nih.gov/projects/gap/cgi-bin/study.cgi?id=phs000007., we provide a full disclosure of the totality of our results, including all "non-significant" associations. In light of the multiple association tests that result from a GWAS, a powerful and efficient follow-up approach to increase the statistical confidence for an association of common variants with complex traits is a staged design, in which a modest number of results from the first stage are then tested in a second independent sample and the combined statistical evidence considered [36]. A further approach may be to seek evidence of *in silico *replication of our initial findings in other GWAS studies. The latter approach will be feasible with completion of the planned dense GWAS (the NHLBI's SNP Health Association [SHARe] Study) in over 9,000 men and women from the Framingham Heart Study [37].

## Abbreviations

AAC = abdominal aortic calcium; ABI = ankle-brachial index; CAC = coronary artery calcium; FBAT = family-based association test; GEE = generalized estimating equations; GWAS = genome-wide association study; IMT = intimal-media thickness; LOD = logarithm of the odds; MDCT = multidetector computed tomography; SCA = subclinical atherosclerosis; SNP = single-nucleotide polymorphism.

## Competing interests

The authors declare that they have no competing interests.

## Authors' contributions

CJO conceived of the MDCT project, planned the analyses, and drafted and critically revised the manuscript. LAC assisted in planning andconducting the analyses, and inwriting and critically revising the manuscript. RBD assisted in planning the carotid measurements and contributed to critically revised the manuscript. CSF assisted in planning andconducting the analyses, and inwriting and critically revising the manuscript. UH planned the MDCT project and assisted in writing and critically revising the manuscript. SJH planned and conducted the analyses. EI critically revised the manuscript. CYL assisted in the analysis and critically revised the manuscript. JMM conceived of the ABI measurement project and contributed to drafting and critically revising the manuscript. JFP contributed to conceiving the carotid measurement project and contributed to critically revising the manuscript. PAW contributed to conceiving the carotid measurement project and contributed to critically revising the manuscript. SD planned and conducted the analyses, and drafted and critically revised the manuscript.
